# Ultrasound-Driven enhancement of Pt/C catalyst stability in oxygen reduction reaction

**DOI:** 10.1016/j.ultsonch.2023.106730

**Published:** 2023-12-17

**Authors:** Hyunjoon Lee, Eunbi Park, Eunjik Lee, Iksung Lim, Tae-Hyun Yang, Gu-Gon Park

**Affiliations:** a Fuel Cell Laboratory, Korea Institute of Energy Research (KIER), Daejeon 34129, Republic of Korea; b Graduate School of Energy Science and Technology (GEST), Chungnam National University, 99 Daehak-ro, Yuseong-Gu, Daejeon 34134, South Korea; c Department of Energy Engineering, University of Science and Technology, 217 Gajeong-ro, Yuseong-gu, Daejeon 34113, Republic of Korea

**Keywords:** Oxygen reduction reaction, Polymer electrolyte membrane fuel cells, Platinum catalyst, Ultrasound-assisted polyol synthesis, Stability, Carbon oxygenation

## Abstract

Polymer electrolyte membrane fuel cells (PEMFCs) have reached the commercialization phase, representing a promising approach to curbing carbon emissions. However, greater durability of PEMFCs is of paramount importance to ensure their long-term viability and effectiveness, and catalyst development has become a focal point of research. Pt nanoparticles supported on carbon materials (Pt/C) are the primary catalysts used in PEMFCs. Accomplishing both a high dispersion of uniform metal particles on the carbon support and robust adhesion between the metal particles and the carbon support is imperative for superior stability, and will thereby, advance the practical applications of PEMFCs in sustainable energy solutions. Ultrasound-assisted polyol synthesis (UPS) has emerged as a suitable method for synthesizing catalysts with a well-defined metal-support structure, characterized by the high dispersion and uniformity of metal nanoparticles. In this study, we focused on the effect of ultrasound on the synthesis of Pt/C via UPS and the resulting enhanced stability of Pt/C catalysts. Therefore, we compared Pt/C synthesized using a conventional polyol synthesis (Pt/C_P) and Pt/C synthesized via UPS (Pt/C_U) under similar synthesis conditions. The two catalysts had a similar Pt content and the average particle size of the Pt nanoparticles was similar; however, the uniformity and dispersion of Pt nanoparticles in Pt/C_U were better than those of Pt/C_P. Moreover, *ex/in-situ* analyses performed in a high-temperature environment, in which nanoparticles tend to agglomerate, have revealed that Pt/C_U exhibited a notable improvement in the adhesion of Pt particles to the carbon support compared with that of Pt/C_P. The enhanced adhesion is crucial for maintaining the stability of the catalyst, ultimately contributing to a better durability in practical applications. Ultrasound was applied to the carbon support without the Pt precursor under the same UPS conditions used to synthesize Pt/C_U to identify the reason for the increased adhesion between the Pt particles and the carbon support in Pt/C_U, and we discovered that oxygen functional groups (C-O, C = O, and O-C = O) for anchoring site of Pt particles were generated in the carbon support. Pt/C_U displayed an increase in stability in an electrochemical accelerated stress test (AST) in an acidic electrolyte. The physical and chemical effects of ultrasound on the synthesis of Pt/C via UPS were identified, and we concluded that UPS is suitable for synthesizing carbon supported electrocatalysts with high stability.

## Graphical abstract

**Figure:**
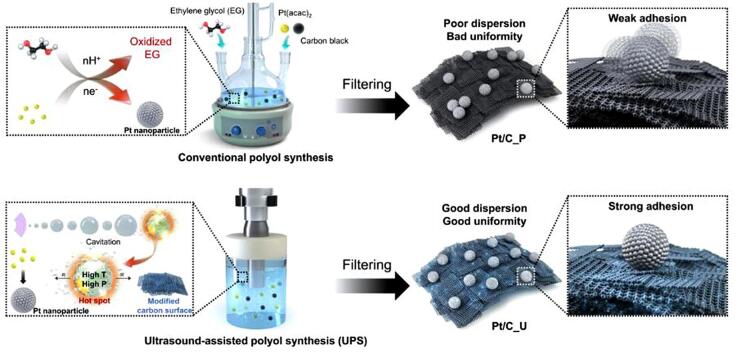

## 1 Introduction

In recent years, as the climate crisis has become more acute, polymer electrolyte membrane fuel cells (PEMFCs) which do not produce carbon emissions have been proposed as a promising alternative to conventional energy sources [1], [2]. PEMFCs have already been used in automobiles and have entered the commercialization stage. Recently, PEMFCs have been considered for use in commercial heavy-duty vehicles (bus, truck, etc.) [2], [3], [4]. Therefore, the durability of PEMFCs is becoming more important. Currently, Pt/C catalysts consisting of Pt nanoparticles supported on carbon materials are commonly used materials for the oxygen reduction reaction (ORR) in PEMFCs, and the demand for durable catalysts is increasing [5], [6]. Although Pt alloy catalysts are expected to be applied in passenger cars, research is still focused on Pt/C catalysts due to the need for long-term stability, especially in heavy-duty vehicles. However, the performance of these Pt catalysts is reduced under fuel cell operation conditions because the high surface energy of the Pt nanoparticles and their weak bonding to the carbon support causes the nanoparticles to agglomerate and detach from the carbon support [7], [8], [9]. Therefore, to guarantee the stability of fuel cell catalysts, it is important to develop a catalyst synthesis method that can improve the dispersion of small and uniform metal particles on the support and increase the binding strength between metal particle and carbon support [10]. One method that has been used to date is polyol synthesis.

Polyol synthesis is a promising method because it allows for the relatively easy synthesis of large quantities of uniform, small particles that are suitable for use as catalysts for fuel cells [11], [12], [13]. A liquid organic compound such as ethylene glycol (EG), which is typically used in polyol synthesis methods, acts as both a solvent and reducing agent. There are several advantages associated with this set up. The high boiling point of the liquid organic compound ensures crystalline particles are obtained. The synthesized metal particles are not oxidized, and the structure of the particles is controlled by the high viscosity of the liquid organic compound which regulates diffusion in the growth of the particles. In addition, particle agglomeration is mitigated because the liquid organic compound coordinates with the surface of the particles as well as the metal precursor [13]. Furthermore, recent researchers have utilized various modified polyol methods which include using two or more polyol solutions with different viscosities simultaneously, adding additives, or using pulsed microwave [12], [14], [15], [16], [17], [18], [19]. Among the modified polyol methods, ultrasound-assisted polyol synthesis (UPS) has been widely used to prepare uniform nanocrystalline materials due to its unique synthesis mechanism. Moreover, the ultrasound used in UPS has the advantage of suppressing the agglomeration of reactants and reducing the size of the nanoparticles during synthesis. In addition, excellent research results have been reported on the one-pot synthesis of complex alloys such as core–shell structures by applying UPS [20], [21], [22], [23].

Ultrasound has been applied to nanoparticle synthesis until recently due to its unique properties. During ultrasonic irradiation of a liquid, the bubbles are subjected to repeated expansion and compression by the ultrasonic wave and eventually collapse to form cavities, creating hot spots with temperatures as high as 5000 K, pressures of 1000 atm, and a fast cooling rate of 10^9^ K/s [24]. These extraordinary conditions impart unique physical and chemical features to the synthesis process and have been used to modify the surface of the support materials or enhance the dispersion of the nanoparticles, particularly in the synthesis of electrocatalysts [20]. For example, in the treatment of acid-treated carbon nanotubes using ultrasound, highly dispersed Pt nanoparticles formed on the support because they were prevented from agglomerating during the treatment process [25]. In addition, studies have been reported in which ultrasound was applied to the impregnation, micro-emulsion, and polyol methods to prevent agglomeration of reduced Pt nanoparticles to synthesize a catalyst with high particle dispersion [26], [27], [28], [29]. However, it has been fairly difficult to clearly show the effect of using ultrasound during catalyst synthesis because previous studies have focused solely on the dispersion enhancement by ultrasound application, using only transmission electron microscopy (TEM) images or the studies have compared catalysts made by different synthesis methods or materials [26], [27], [28].

In this study, we focused on improving the stability of catalysts by ultrasound in UPS and attempted to understand the effects of ultrasound on catalyst stability. We synthesized Pt/C with Pt nanoparticles on Vulcan XC-72 (VXC72), the most commonly used carbon support, using two methods which were matched as closely as possible in terms of synthesis conditions (e.g., amount and type of carbon, precursors, and reaction time) and the amount of Pt in the catalyst. The two catalysts were named Pt/C_P (synthesized by the conventional polyol synthesis) and Pt/C_U (synthesized via UPS). TEM, X-ray diffraction (XRD), and *in-situ* TEM analysis showed that the Pt nanoparticles in Pt/C_U were more evenly distributed and uniform, and the Pt nanoparticles did not grow larger than those in Pt/C_P even at high temperatures (800 °C). In addition, to determine the cause of the improvement in the thermal stability of the catalyst by ultrasound, various analyses of VXC72 which was irradiated with ultrasound under the same conditions of synthesizing Pt/C_U revealed that the surface of carbon was oxygenated. Therefore, the improvement in the stability of the Pt nanoparticles was ascribed to the oxygenation of the carbon surface. The functionalized surface of carbon acts as a nucleation and anchoring site, which improves the uniformity and dispersion of the Pt nanoparticles, and enhances the binding with the Pt nanoparticles [10], [30], [31], [32], [33], [34], [35], [36], [37]. Pt/C_U, which displays the improved stability of Pt nanoparticles on the carbon support due to the influence of ultrasound, showed higher stability than Pt/C_P in the electrochemical stability test under acidic electrolyte. The effects of ultrasound on the synthesis via UPS identified in this study are not only physical effects that can synthesize a catalyst with less EG than that accomplished by the conventional polyol synthesis because of fast mass transport and improve the uniformity and dispersion of the formed Pt nanoparticles by suppressing the agglomeration phenomenon of the reaction product [20], but also chemical effects on the surface of carbon support, and these results are expected to contribute to the future synthesis of carbon supported electrocatalysts that require long-term stability.

## 2 Experimental

### 2.1 Synthesis of Pt/C by conventional polyol synthesis

Pt(acac)_2_ (0.823 g) and 1,600 mL of N_2_ saturated ethylene glycol (EG) were mixed in a three-neck round bottomed flask. NaOH (0.1 M) was added to the perfectly mixed Pt precursor solution to adjust the pH to 11. Thereafter, 0.6 g of VXC72 was mixed with the Pt precursor solution by stirring with a magnetic stirring bar for 1 h followed by 30 min sonication in a sonic bath (HWASHIN INSTRUMENT, Powersonic 620). Thereafter, the precursor dispersion was heated to 160 °C for 3 h in a heating mantle to reduce the Pt precursor to Pt nanoparticles. The synthesized Pt/C was obtained by filtering with a membrane filter (90 mm diameter, 1.0 μm pore size). The synthesized Pt/C was repeatedly washed with excess ethanol and deionized water during filtering to remove residual EG. The obtained Pt/C was dried in a vacuum oven at 60 °C for 24 h. The synthesized Pt/C was denoted as Pt/C_P.

### 2.2 Synthesis of Pt/C by ultrasound-assisted polyol synthesis

Pt(acac)_2_ (0.823 g), 0.6 g of VXC72 and 60 mL of N_2_ saturated EG were mixed in a 100 mL glass vial. The vial containing the precursor dispersion was connected to a horn-type sonicator (Sonic & Materials, VCX 750 model, tip diameter: 13 mm, solid probe) using an in-house Teflon connector. Thereafter, it was irradiated at a 40 % amplitude (300 W) for 3 h. The synthesized Pt/C was obtained by filtering with a membrane filter (90 mm diameter, 1.0 μm pore size). The synthesized Pt/C was repeatedly washed with excess ethanol and deionized water during filtering to remove residual EG. The obtained Pt/C was dried in a vacuum oven at 60 °C for 24 h. The synthesized Pt/C was denoted as Pt/C_U.

### 2.3 Characterization

Thermogravimetric analysis (TGA) was performed using a Scinco TGA-N 1000 thermal analyzer. The heating rate was 5 °C min^−1^ and the temperature range was 25–800 °C under a constant air flow of 5 mL min^−1^. High resolution-transmission electron microscopy (HR-TEM) images were obtained using a JEM-2100F HR (JEOL Ltd.) instrument. The acceleration voltage used for HR-TEM was set at 200 kV. Powder XRD patterns were obtained using a Rigaku SmartLab X-ray diffractometer with Cu Kα radiation at a scan rate of 5° min^−1^. A Nexsa G2 instrument (Thermo Fisher Scientific) equipped with a microfocused and monochromated Al K-Alpha energy source was used for X-ray photoelectron spectroscopy (XPS). The *in-situ* TEM was investigated to observe the change in the particle size distribution of Pt nanoparticle supported on carbon using FE-TEM (JEM-F200, JEOL Ltd.) at an accelerating voltage of 200 kV. The observations were performed by loading the sample onto a Fusion Thermal E-chips (E-FHDC-VO-10, Protochips Inc., USA), which was placed in a heating holder (Fusion, Protochips Inc.). The sample was heated from 25 to 800 °C at a heating rate of 30 °C min^−1^. Elemental analysis data for C, H, N, S, and O were obtained using an elemental analyzer (EA, FLASH 2000 series, Thermo Scientific). Fourier transform infrared (FT-IR) spectra were recorded using a Nicolet FT-IR spectrometer (MAGNA-IR 560) with KBr pellets. The dispersion stability in ethanol of the sample dispersion was characterized using a LUMiSizer (LS610, L.U.M. GmbH, Germany). The instability index is calculated using the equation provided [38].

$$\frac{\Delta T_{i}}{\Delta T_{\max}}=\frac{\sum_{j=r_{\min}}^{r_{\max}}T_{i,j}^{\mathrm{diff}}}{\left({\overset{¯}{T}}_{\mathrm{End}}-{\overset{¯}{T}}_{1}\right)∙\left(j_{r_{\max}}-j_{r_{\min}}\right)},$$

where $T_{i,j}^{\mathrm{diff}}$ represents the variance between the initial and subsequent transmission profiles, ${\overset{¯}{T}}_{\mathrm{End}}$ indicates the difference between the average transmission of a cell with only ethanol and ${\overset{¯}{T}}_{1}$, which represents the average transmission of the initial profile. The value $j_{r_{\max}}-j_{r_{\min}}$ corresponds to the number of transmission values within the position range spanning from the maximum to the minimum.

### 2.4 Electrochemical analysis

A potentiostat (BioLogic, VSP-3e) with a rotating disk electrode was used to perform cyclic voltammetry (CV) and linear sweep voltammetry (LSV) for electrochemical characterization. The experimental setup consisted of a three-electrode electrochemical cell configuration, where a Pt coiled wire served as the counter electrode, a reversible hydrogen electrode (RHE) (Gaskatel GmbH, Germany) served as the reference electrode, and a glassy carbon (GC) electrode with a geometric area of 0.196 cm^2^ served as the working electrode. The working electrode was coated with the catalyst for testing. To prepare the working electrode, a catalyst ink was created by mixing the catalyst (10 mg) and Nafion® D-521 (5 μL) in a 2 mL solution containing de-ionized water and isopropyl alcohol (IPA) in a 4:1 vol ratio. The mixture was sonicated. The catalyst ink was applied to the GC electrode, to generate a Pt content of 0.02 mg_pt_ cm^−2^. CV spectra were recorded within a scan range of 0.03–1.1 V (vs. RHE) in N_2_-saturated HClO_4_ (0.1 M) at a scan rate of 20 mV s^−1^. For LSV, the scan range was set from 0.05 to 1.1 V (vs. RHE) with a scan rate of 10 mV s^−1^ in O_2_-saturated HClO_4_ (0.1 M) at a rotating speed of 1600 rpm. To correct the LSV curves for ORR, iR-compensation was applied, and the background capacitance current was removed. For the accelerated stress test (AST) for the electrocatalyst, chronoamperometry was performed at 0.6 and 0.95 V for 3 s.

## 3 Results and discussion

### 3.1 Synthesis of Pt/Cs via conventional polyol or ultrasound-assisted polyol synthesis

**Fig. 1:**
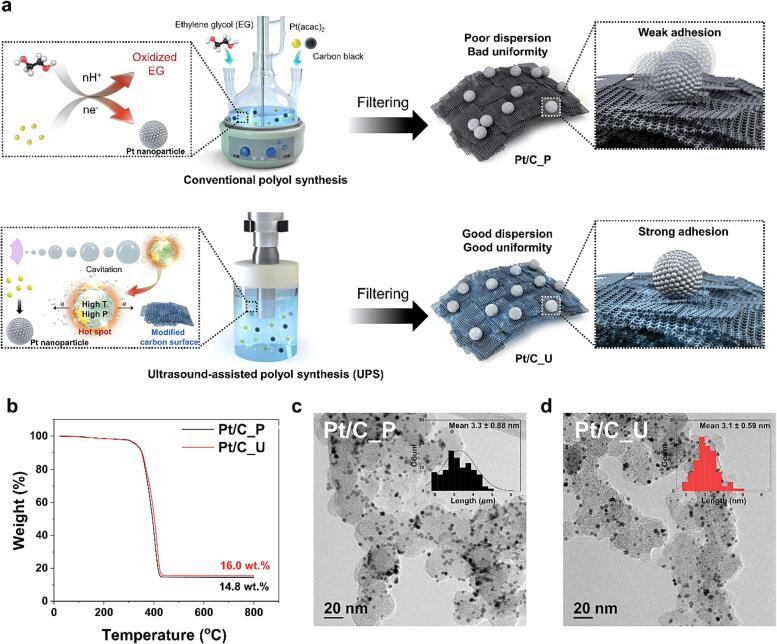
Synthesis of Pt/C by conventional polyol synthesis or ultrasound-assisted polyol synthesis. (a) Schematic illustration of synthesis of Pt/C_P and Pt/C_U. (b) Thermogravimetric analysis profiles, (c, d) Transmission electron microscopy images, and size distribution of Pt/C_P and Pt/C_U.

To determine the effect of ultrasound on the synthesis of Pt/C catalysts, it is important to match the synthesis conditions (type and amount of carbon, Pt precursors, and reaction time) and Pt amount for Pt/C_P synthesized by conventional polyol synthesis method and Pt/C_U synthesized by UPS to minimize other variables. Fig. 1a shows the process of synthesizing Pt/C by each method. (Details are provided in the experimental section) Pt/C_P was synthesized by adding Pt precursor and carbon support in EG, adjusting the pH to 11 and heating with a mantle heater, and was obtained after filtration. The synthesized Pt/C_P was used as a comparison to determine the effect of ultrasound on the synthesis. Pt/C_U was obtained by adding a Pt precursor and carbon support to EG, applying ultrasonic waves, and then filtering the Pt/C_U through a filter.

The synthesis of the catalyst using UPS was relatively simple compared with that of the conventional polyol synthesis as UPS does not require pH adjustment and the quantity of EG can be significantly reduced. The main difference between polyol synthesis and UPS is the manner of reducing the Pt precursor. For the polyol synthesis, when the temperature increases, EG acts as a reducing agent to reduce the Pt precursor, resulting in Pt nanoparticles. However, in UPS, EG is oxidized by heat to reduce the Pt precursor as in conventional polyol synthesis; additionally, via hot spots created by ultrasound-induced cavitation, EG becomes a primary radical; the secondary radicals induced by this primary radical reduce the Pt precursor to form nanoparticles [30]. TGA was performed in an air atmosphere to determine the Pt content of each synthesized catalyst. Fig. 1b shows the TGA results of each catalyst. The Pt content of each catalyst was found to be 16.0 wt% for Pt/C_P and 14.8 wt% for Pt/C_U. That is, the Pt/C catalysts prepared by each synthesis method have similar Pt contents. When the same carbon is used for synthesis, the Pt content affects the distance between the Pt particles, therefore, it is assumed that there is no variable in particle spacing between the two catalysts that is dependent on the Pt content. Fig. 1c and d are TEM images of the Pt/C_P and Pt/C_U, respectively. For both catalysts, the synthesized Pt nanoparticles were supported on the carbon support (VXC72). The particle size of each catalyst is 3.3 nm for Pt/C_P and 3.1 nm for Pt/C_U, which are similar, but the size distribution for Pt/C_U is narrower than that for Pt/C_P and the dispersion of Pt nanoparticles for Pt/C_U is improved.

**Fig. 2:**
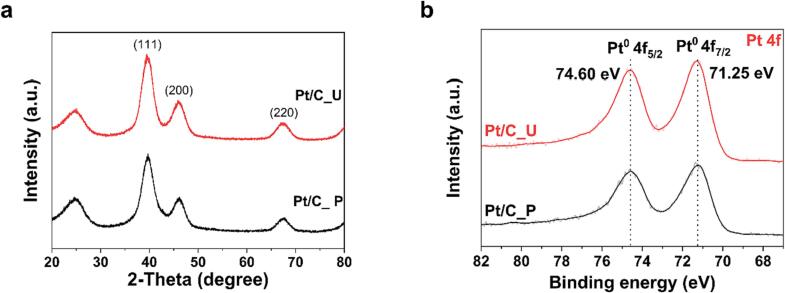
Characterization of Pt/C_P and Pt/C_U with X-ray analysis. (a) X-ray diffraction and (b) X-ray photoelectron spectroscopy spectra of Pt/C_P and Pt/C_U.

X-ray analysis was performed to characterize the Pt particles synthesized on each catalyst. Fig. 2a shows the XRD spectra of each Pt/C. Both Pt/C catalysts have the same peaks of the face-centered cubic (fcc) structure. Using the Scherrer equation to calculate the crystallite size of each catalyst based on the (1 1 1) peak, it was determined that the crystallite size of Pt/C_P was 2.4 nm and that of Pt/C_U was 2.7 nm. The results of the XRD analysis are summarized in Table S1. XPS analysis was performed to determine the oxidation state of the synthesized Pt particles (Fig. 2b). The Pt nanoparticles of each Pt/C catalyst synthesized by both methods existed as metallic Pt^0^. In summary, both the conventional polyol and UPS methods are considered to reduce the Pt precursor equally for synthesizing Pt/C, but the ultrasound used in UPS has a positive effect on the dispersion and uniformity of the Pt nanoparticles on the carbon support.

### 3.2 Comparing thermal stability of Pt/C_P and Pt/C_U

**Fig. 3:**
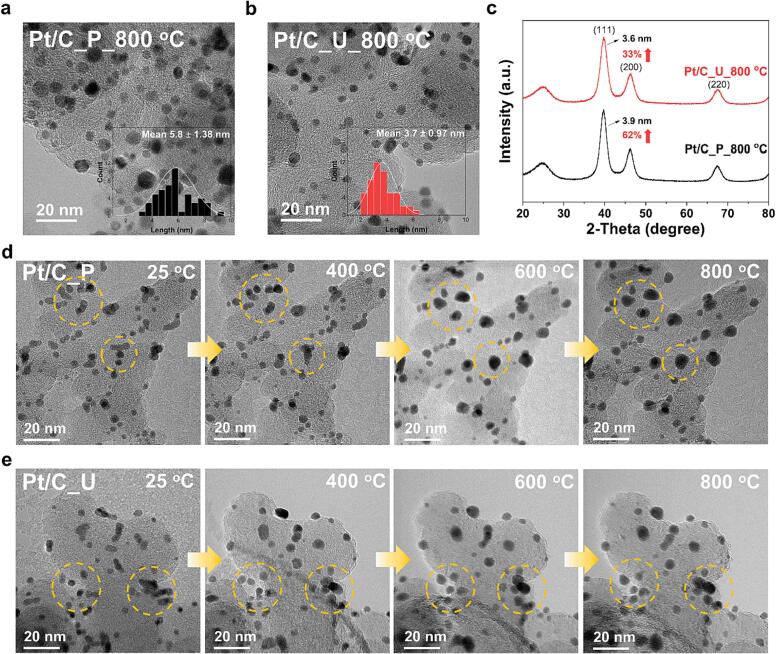
Comparing stability of Pt/C_P and Pt/C_U in a high temperature environment. (a, b) Transmission electron microscopy images, size distribution, and (c) X-ray diffraction spectra of Pt/C_P_800 ^o^C and Pt/C_U_800 ^o^C. (d, e) Transmission electron microscopy images of Pt/C_P and Pt/C_U during *in-situ* heating.

TEM analysis confirmed the improved uniformity and dispersion of Pt particles in Pt/C_U, but since TEM analysis has some limitations, further analysis was required. Therefore, we exposed each catalyst to high temperatures, an environment in which nanoparticles tend to aggregate, to determine their stability. Since each synthesized catalyst has the same particle size and amount of Pt, it is believed that the thermal stability test will be able to determine the dispersion and uniformity of the Pt particles and the difference in adhesion between the carbon support and the Pt nanoparticle. Pt/C_P and Pt/C_U were annealed in a furnace at 800 °C in a mixed gas (N_2_ gas with 5 % H_2_) atmosphere for 2 h. Fig. 3a and b show the TEM images and particle size distribution results of each catalyst after annealing.

For Pt/C_P, agglomerated Pt particles are clearly visible after annealing (Pt/C_P_800 °C), while for Pt/C_U, small particles that are not yet agglomerated after annealing (Pt/C_U_800 °C) are well-dispersed. Comparing the size distributions, Pt/C_P_800 °C has an average particle size of 5.8 nm while Pt/C_U_800 °C has an average particle size of 3.7 nm and the size distribution of Pt/C_U_800 °C is definitely narrower. Similar results were found in the XRD results (Fig. 3c), where the crystallite size of each catalyst was calculated based on the peak (1 1 1) peak using the Scherrer equation; the crystallite size of Pt/C_P_800 °C was 3.9 nm, a 62 % increase in crystallite size compared with that before heat treatment, while that of Pt/C_U_800 °C was 3.6 nm, a 33 % increase. These annealing test results are a direct indication of the improved stability of Pt/C_U and can be attributed to the improved dispersion of Pt nanoparticles in Pt/C_U, which was identified in the previous analysis. To more directly determine the stability of the Pt particles in an environment of high temperature, each catalyst was then analyzed by TEM with *in-situ* heating. The heating condition was a fast-ramping rate of 30 °C min^−1^ from 25 °C to 800 °C under vacuum, and the temperature was maintained at 800 °C for approximately 7 min. In particular, the *in-situ* analysis focused on the difference between the adhesion of particles and carbon support for the catalysts prepared using the two synthesis techniques, which was difficult to identify in previous analyses. Therefore, the analysis was performed by specifying areas on each catalyst where Pt particles were physically close to each other. Fig. 3d and e are TEM images of Pt/C_P and Pt/C_U at 25 °C, 400 °C, 600 °C, and 800 °C. Particular differences between the two catalysts are marked with yellow circles. For Pt/C_P, we first observed that at 400 °C, the particles that were close to each other started to agglomerate into a single particle; at 600 °C, the agglomerated particles were fully agglomerated and grew into large rounded particles; and at 800 °C, the particles were already large enough that we could not observe any more changes. Pt nanoparticles in Pt/C_U, by contrast, were in close proximity to each other at 400 °C, but not agglomerated or completely coalesced, and remained as individual particles with little change at higher temperatures. There was a marked difference in the particle size evolution of each catalyst at 25 °C and 800 °C, with the initial particle size of Pt/C_P averaging 3.5 nm and increasing to 5.4 nm at 800 °C, and the initial particle size of Pt/C_U averaging 3.0 nm and increasing marginally to 3.9 nm at 800 °C (Fig. S1). The size distribution at 800 °C is much narrower for Pt/C_U.

The agglomeration of supported nanoparticles at elevated temperatures is influenced by the physical distance between the particles and their adhesion to the support. In this analysis, we intentionally analyzed that part where the distance between the particles was close, so we consider that the difference in adhesion to the carbon support is more dominant than the physical distance between the nanoparticles. Therefore, TEM with *in-situ* heating analysis showed that the adhesion between Pt nanoparticles and carbon support in Pt/C_U was better than that in Pt/C_P. In summary, the thermal stability test confirmed that the stability of Pt/C_U is superior to that of Pt/C_P, although the catalysts contain the same amount of Pt. This stability improvement suggests that the uniformity and dispersion of Pt nanoparticles were improved due to the influence of ultrasound in the synthesis of Pt/C_U, which physically prevents the agglomeration of reactants and reaction products [20], and that ultrasound may have affected the carbon support, causing changes in the surface of carbon and improving the adhesion of Pt particles.

### 3.3 Effect of ultrasound on carbon support during ultrasound-assisted polyol synthesis

The interaction between the support material and the metal nanoparticles is highly dependent on the surface state of the support, therefore, to increase the performance of the catalyst, many methods are used to tailor the interaction between the metal nanoparticles and the support by doping other elements on the surface of the support material or via functionalization [10]. Therefore, to understand the effect of ultrasound on the surface of carbon when synthesizing Pt/C_U, VXC72, used as a carbon support under the same conditions but excluding the Pt precursor, was irradiated with ultrasound (VXC72_U) and analyzed. EA analysis of VXC72 with and without ultrasonic irradiation was performed and the results are shown in Table 1.

**Table 1:** Elemental analysis results of VXC72 and VXC72_U.

Sample	C(%)	H(%)	N(%)	S(%)	O(%)	Total
VXC72	98.93	0.23	0.05	0.48	0.31	100
VXC72_U	96.94	0.41	0.05	0.48	2.12	100

The EA analysis showed that VXC72_U had a notable increase in the ratio of O, that is, 6.8 times higher than that of VXC72. The ultrasound irradiation may have induced oxygen-related functional groups in VXC72. Hydroxyl (OH), carbonyl (C = O), and carboxyl (O-C = O) groups are generated on the surface of carbon by applying microwaves with EG to carbon materials for functionalization of carbon materials [39], [40]. For more direct confirmation, FT-IR analysis was performed to confirm the presence of oxygen functional groups on VXC72_U.

**Fig. 4:**
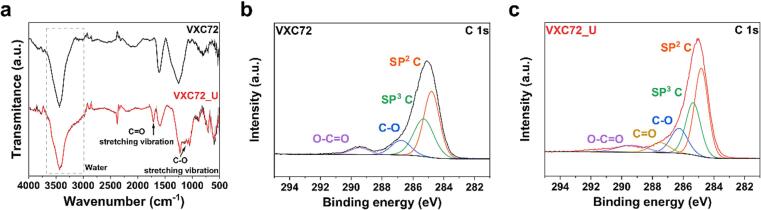
Characterization of VXC72 and VXC72_U for confirming effect of ultrasound on carbon support during UPS. (a) Fourier transform infrared spectra and (b, c) C 1 s X-ray photoelectron spectroscopy spectra.

Fig. 4a shows the FT-IR spectra of VXC72 and VXC72_U. Compared with the FT-IR spectrum of VXC72, peaks related to oxygen functional groups in the FT-IR spectrum of VXC72_U were characterized. A C-O (stretching vibration) peak at 1160 cm^−1^ and a C = O (stretching) peak at 1700 cm^−1^, which were not observed in VXC72, were identified in VXC72_U [36], [39], [41], [42]. In addition, a broad O-H (stretching vibration) band in adsorbed water at 3000–3680 cm^−1^ in VXC72_U was broadened [36], [41], [42], [43]. These results directly indicate the generation of oxygen functional groups in VXC72_U due to ultrasound irradiation. Identifying the peaks of the other functional groups proves challenging due to the overlapping and indistinguishable nature of their signals on both carbons [36]. Additional XPS analysis was performed to support the FT-IR analysis. Fig. 4b and c are the C 1 s XPS spectra of VXC72 and VXC72_U, respectively. The C 1 s peaks of VXC72 are separated into C-C(sp2), C-C(sp3), C-O and O-C = O, with the area percentages occupied by each peak being 47.2 %, 34.4 %, 12.7 %, and 9.6 %, respectively [36], [44]. The C 1 s peaks of VXC72_U are separated into C-C(sp2), C-C(sp3), C-O, C = O, and O-C = O, with the area percentages occupied by each peak being 42.9 %, 27.9 %, 15.0 %, 6.9 %, and 9.6 %, respectively [36], [44]. The XPS results showed that the ratio of the areas of both C-O and O-C = O increased in VXC72_U, and C = O was identified, which was not present in VXC72. These results are consistent with the FT-IR results. The XPS results are summarized in Table 2.

**Table 2:** C 1 s X-ray photoelectron spectroscopy peak fitting data of VXC72 and VXC72_U.

Bond (Binding energy)	VXC72 (area %)	VXC72_U (area%)
C-C (sp^2^) (284.8 eV)	47.2	42.9
C-C (sp^3^) (285.3 eV)	34.4	27.9
C-O (286.8 eV)	12.7	15.0
C = O (287.5 eV)	–	6.9
O-C = O (289.3 eV)	5.7	7.3

To investigate the effect of oxygen functional groups on the surface of carbon caused by ultrasound irradiation, TGA analysis was performed in an air atmosphere (Fig. S2), and it can be seen that VXC72_U is decomposed earlier than VXC72, indicating that the number of defect sites containing oxygen functional groups in VXC72_U is increased [45]. Furthermore, since the oxygen functional groups are hydrophilic, we used a LUMiSizer to check the dispersion retention in ethanol (Fig. S3) and discovered that the instability index, a parameter indicating dispersion over time, remained markedly lower for VXC72_U compared with that of VXC72 [38]. Considering all the previous analyses, it is clear that ultrasound-induced oxygen functional groups on the surface of carbon during UPS. The oxygen functional groups on the surface of carbon can be used as nucleation and anchoring sites for Pt nanoparticles to grow, which affects the improvement of the uniformity and dispersion of Pt nanoparticles in Pt/C synthesis, and increases adhesion with the reduced Pt nanoparticles [10], [36]. Therefore, these results indicate that the physical and chemical effects of ultrasound are directly responsible for the increased stability of Pt/C_U in the previous annealing tests.

### 3.4 Electrochemical analysis for confirming improved stability of Pt/C_U

The previous analyses indicated that the stability of Pt/C_U increased because the dispersion and uniformity of Pt nanoparticles and the adhesion between Pt nanoparticles and carbon support improved. Therefore, a half-cell AST was performed to confirm the improved stability of electrocatalyst in acid electrolyte. The AST was performed by square-wave cycling between 0.6 V (3 s) and 0.95 V (3 s) for 30,000 (30 k) cycles, according to the US DOE protocol for electrocatalysts. Thereafter, the stability of each catalyst was evaluated by comparing the beginning of life (BOL) and end of life (EOL) performance.

**Fig. 5:**
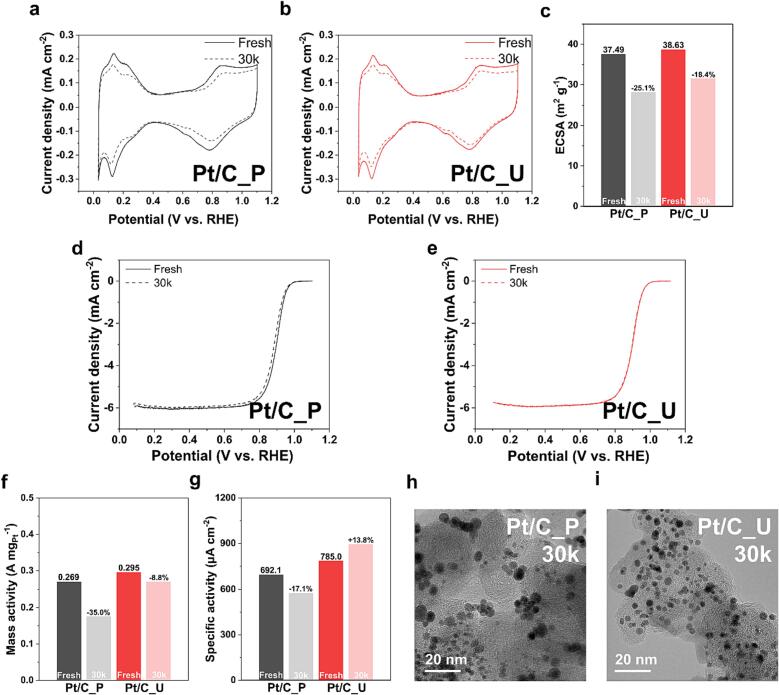
Stability analysis of Pt/Cs. (a, b) Cyclic voltammetry curves, (c) electrochemical active surface area, (d, e) linear sweep voltammetry curves, (f) mass activities, and (g) specific activities of Pt/C_P and Pt/C_U, before and after 30 k cycles of accelerated stress test. (h, i) Transmission electron microscopy images of Pt/C_P and Pt/C_U after 30 k cycles of accelerated stress test.

Fig. 5a and b show the CV results before and after the AST of Pt/C_P and Pt/C_U.

The electrochemical active surface area (ECSA) was calculated using the hydrogen desorption area of CV; Pt/C_P had an ECSA of 37.49 m^2^/g and that of Pt/C_U was 38.63 m^2^/g. The values are not dissimilar because the average size of the particles is similar, but Pt/C_U has a slightly larger value because the uniformity and dispersion of the particles are better than those of Pt/C_P. However, after AST 30 k cycles, there was a notable difference in the ECSA (Fig. 5c). The ECSA of Pt/C_P decreased by 25.1 % compared with that of the initial ECSA, while the ECSA of Pt/C_U decreased by only 18.4 %. These results show that Pt/C_U displays better stability than Pt/C_P under the conditions of the AST 30 k cycles. Comparing the LSV results to compare ORR activity, we discovered that the initial half-wave potential (E_1/2_) was also not significantly different, but Pt/C_U displayed a E_1/2_ of 900 mV, which was approximately 3 mV better than that of Pt/C_P (Fig. 5d and e). However, after the AST 30 k cycles, Pt/C_P exhibited a decrease in E_1/2_ of 12 mV, while Pt/C_U exhibited a decrease of only 2 mV, indicating that the decrease was negligible.

Based on the electrochemical analysis results, mass activity and specific activity, which are important indicators of the performance of the catalyst, were calculated before and after the AST, and the results are shown in Fig. 5f and g. The mass activity of Pt/C_U was discovered to be superior to that of Pt/C_P with only an 8.8 % decrease after AST compared with that of the initial performance. The specific activity of Pt/C_U increased by 13.8 % after the AST, and the increase is attributed to the almost unchanged ORR activity despite the decrease in ECSA. TEM analysis was performed after stability testing to directly confirm the improved stability of Pt/C_U in electrochemical activity evaluation, and the results are shown in Fig. 5h and i. The difference between Pt/C_P and Pt/C_U can be clearly seen in the TEM analysis, where the size of the Pt particles in Pt/C_P increased and there were many places where the nanoparticles clustered together. By contrast, in Pt/C_U, the size of the particles did not increase and the distribution of the particles was well maintained. In conclusion, as shown in the previous analysis, the effect of ultrasound during synthesis increased the dispersion of Pt particles in Pt/C_U and improved the adhesion to the carrier, which increased the stability, and this effect can be clearly seen in the actual electrochemical test. The difference between Pt/C_P and Pt/C_U can be clearly seen in the TEM analysis, where the size of the Pt particles in Pt/C_P increased and there were many places where the nanoparticles clustered together, but in Pt/C_U, the size of the particles did not increase notably and the distribution of the particles was well maintained. In conclusion, UPS is feasible compared with the conventional polyol synthesis for catalyst synthesis, and as confirmed by the previous analyses, the effect of ultrasound during UPS increased the dispersion of Pt particles in Pt/C_U and improved the adhesion between Pt nanoparticles and the support, resulting in increased stability, which can be clearly seen in actual electrochemical tests.

## 4 Conclusion

To determine the effect of ultrasound on Pt/C synthesized via UPS and the resulting increase in catalyst stability, we synthesized Pt/C_U via UPS and compared it to Pt/C_P synthesized by the conventional polyol synthesis. Since Pt/C_P and Pt/C_U were synthesized under similar experimental conditions and have a similar Pt content, the effect of ultrasound on Pt/C synthesis could be ascertained. The synthesized Pt/C_P and Pt/C_U had Pt nanoparticles of a similar average size, but TEM analysis showed that the uniformity and dispersion of Pt nanoparticles in Pt/C_U were improved, suggesting the influence of ultrasound during synthesis. Thermal stability tests were performed to determine the differences in the uniformity, dispersion, and adhesion to the Supporting Materials of Pt nanoparticles in Pt/C_U and Pt/C_P. The *in/ex-situ* heating analysis of both catalysts in a high temperature environment in which particles are easily agglomerated showed that the dispersion of Pt nanoparticles in Pt/C_U and the adhesion between Pt nanoparticles and the carbon support increased. To understand the reason for the improvement in the adhesion between the Pt nanoparticles and the carbon support, only the VXC72 used for carbon support was sonicated under the same UPS conditions used to synthesize Pt/C_U and VXC72_U was characterized. FT-IR and XPS results showed that the oxygen functional groups (C-O, C = O, and O-C = O) increased in VXC72_U compared with those in the pristine VXC72. The oxygen functional groups are responsible for the increased dispersion of Pt/C_U and the increased adhesion of Pt particles to the carbon support. The stability of Pt/C_U was compared with that of Pt/C_P by performing electrochemical AST in acidic electrolyte, and it was confirmed that the electrochemical stability increased due to the improved dispersion of Pt nanoparticles and the increased adhesion of Pt nanoparticles to the carbon support. Ultrasound in Pt/C synthesis via UPS physically inhibits the agglomeration of reactants and synthesized particles as previously known, and chemically oxygenates the carbon support, increasing the dispersion of synthesized Pt nanoparticles and their adhesion to the support. The role of ultrasound in catalyst synthesis identified in this study provides a rationale for utilizing ultrasound to synthesize particle-support structure catalysts that require high stability.

## CRediT authorship contribution statement

**Hyunjoon Lee:** Writing – original draft, Investigation, Data curation. **Eunbi Park:** Investigation. **Eunjik Lee:** Writing – review & editing, Investigation. **Iksung Lim:** Investigation. **Tae-Hyun Yang:** Investigation. **Gu-Gon Park:** Writing – review & editing, Funding acquisition.

## Declaration of competing interest

The authors declare the following financial interests/personal relationships which may be considered as potential competing interests: Gu-Gon Park reports financial support was provided by Korea Ministry of Science and ICT. If there are other authors, they declare that they have no known competing financial interests or personal relationships that could have appeared to influence the work reported in this paper.
