# Supplementary material for: Ultrasound-Driven enhancement of Pt/C catalyst stability in oxygen reduction reaction
Source: Ultrason Sonochem. 2023 Dec 17;102:106730. doi: 10.1016/j.ultsonch.2023.106730 (PMC10772287; doi:10.1016/j.ultsonch.2023.106730)
Supplement: Supplementary data 1 [file mmc1.docx]

Supporting Information

^Ultrasound-Driven Enhancement of Pt/C Catalyst Stability in Oxygen Reduction Reaction^

*Hyunjoon Lee^a^, Eunbi Park^a b^, Eunjik Lee^a b c^, Iksung Lim^a b^, Tae-Hyun Yang^a^, and Gu-Gon Park^a b c*^*

^a^ Fuel Cell Laboratory, Korea Institute of Energy Research (KIER), Daejeon 34129, Republic of Korea

^b^ Graduate School of Energy Science and Technology (GEST), Chungnam National University, 99 Daehak-ro, Yuseong-Gu, Daejeon 34134, South Korea

^c^ Department of Energy Engineering, University of Science and Technology, 217 Gajeong-ro, Yuseong-gu, Daejeon 34113, Republic of Korea

^*^Corresponding author.

E-mail address: [gugon@kier.re.kr](mailto:gugon@kier.re.kr)


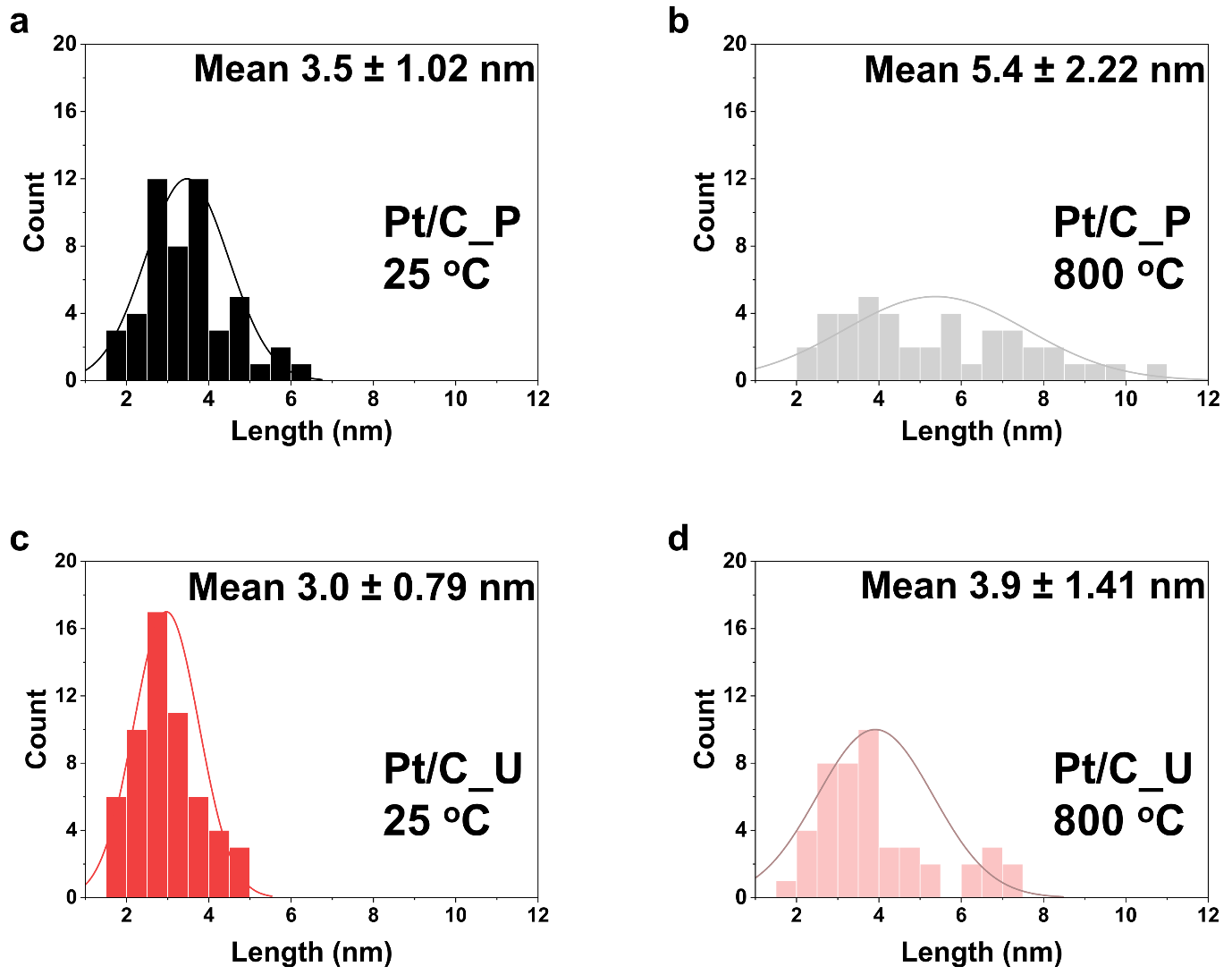


**Fig. S1.** Particle size distribution at 25 °C and 800 °C of (a, b) Pt/C_P and (c, d) Pt/C_U.


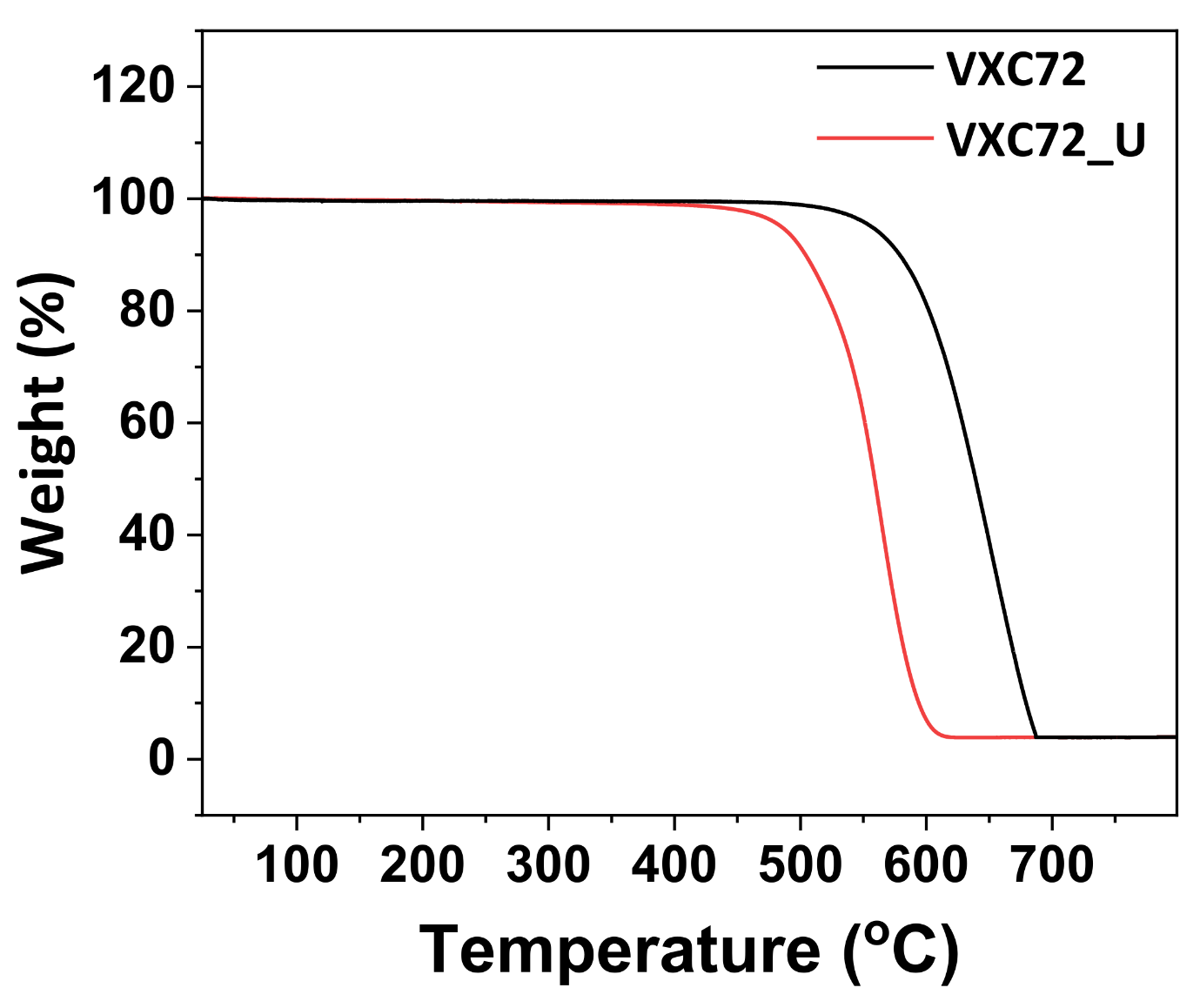


**Fig. S2.** Thermogravimetric analysis profiles of VXC72 and VXC72_U.


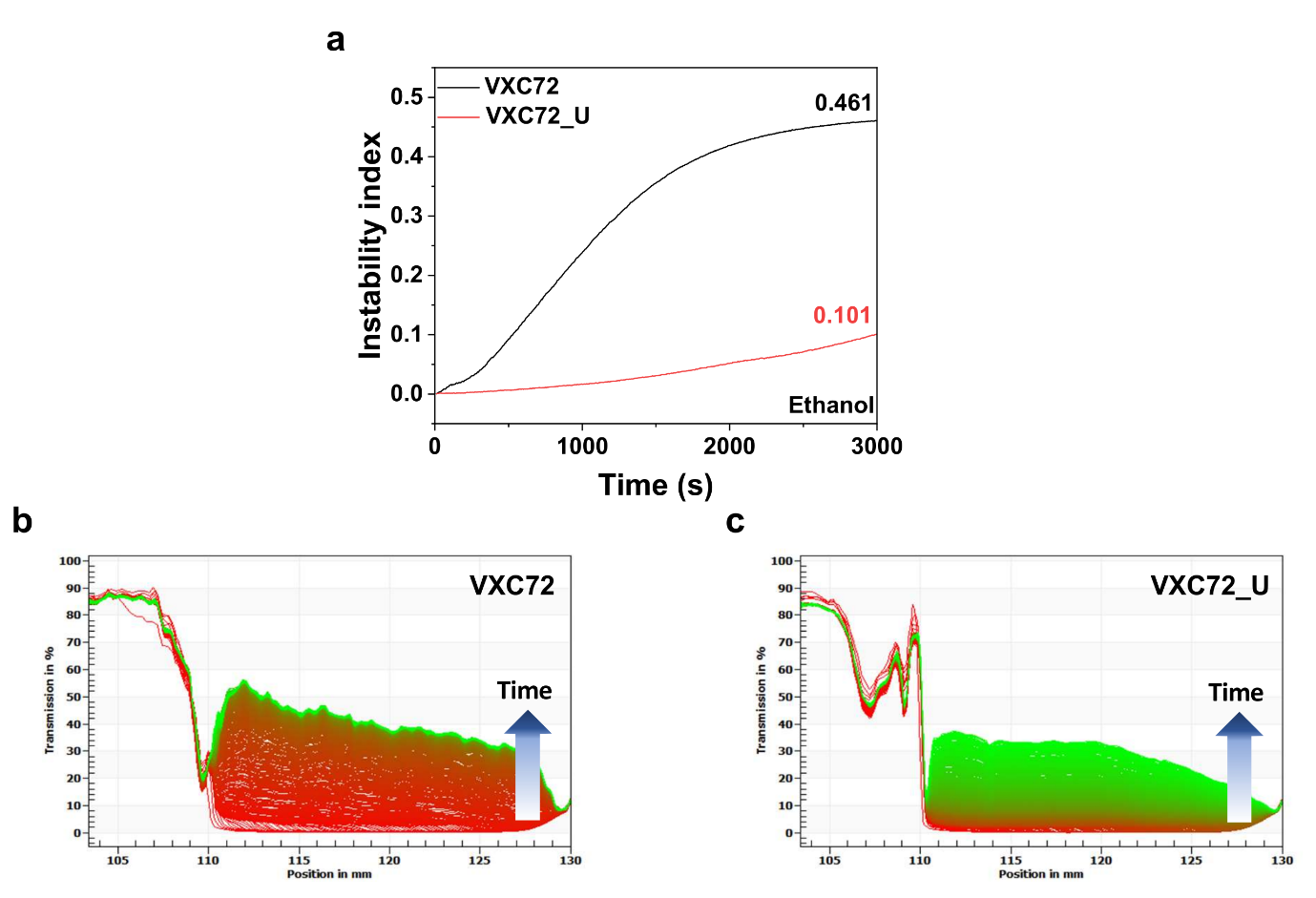


**Fig. S3.** (a) Instability index and (b, c) transmission profiles according to the time for VXC72 and VXC72_U by dispersion stability test in ethanol with LUMiSizer equipment. (0.1 wt% carbon dispersed in ethanol)

**Table. S1** X-ray diffraction peak position of Pt(111) and crystallite size calculated by Scherrer equation based on Pt(111) and Pt(220) peaks of Pt/C_P and Pt/C_U.

| **Sample** | **Peak position**  **(^o^) (111)** | **Crystallite size (nm)** | |
| --- | --- | --- | --- |
|  |  | (111) | (220) |
| **Pt/C_P** | 39.7 | 2.4 | 2.3 |
| **Pt/C_U** | 39.6 | 2.7 | 2.4 |
